# Immune System Evasion as Hallmark of Melanoma Progression: The Role of Dendritic Cells

**DOI:** 10.3389/fonc.2019.01148

**Published:** 2019-11-05

**Authors:** Marco Tucci, Anna Passarelli, Francesco Mannavola, Claudia Felici, Luigia Stefania Stucci, Mauro Cives, Francesco Silvestris

**Affiliations:** Department of Biomedical Sciences and Human Oncology, University of Bari Aldo Moro, Bari, Italy

**Keywords:** melanoma, dendritic cells, microenvironment, checkpoint inhibitors, T-cells

## Abstract

Melanoma is an immunogenic tumor whose relationship with immune cells resident in the microenvironment significantly influences cancer cell proliferation, progression, and metastasis. During melanomagenesis, both immune and melanoma cells undergo the immunoediting process that includes interconnected phases as elimination, equilibrium, and escape or immune evasion. In this context, dendritic cells (DCs) are active players that indirectly counteract the proliferation of melanoma cells. Moreover, DC maturation, migration, and cross-priming as well as their functional interplay with cytotoxic T-cells through ligands of immune checkpoint receptors result impaired. A number of signals propagated by highly proliferating melanoma cells and accessory cells as T-cells, natural killer cells (NKs), tumor-associated macrophages (TAMs), T-regulatory cells (T-regs), myeloid-derived suppressor cells (MDSCs), and endothelial cells participate to create an immunosuppressive milieu that results engulfed of tolerogenic factors and interleukins (IL) as IL-6 and IL-10. To underline the role of the immune infiltrate in blocking the melanoma progression, it has been described that the composition, density, and distribution of cytotoxic T-cells in the surrounding stroma is predictive of responsiveness to immunotherapy. Here, we review the major mechanisms implicated in melanoma progression, focusing on the role of DCs.

## Introduction

Cutaneous melanoma (CM) is an aggressive cancer that arises from melanocytes originating from the neural crest. These cells then migrate into the epidermis, where they undergo maturation and acquire the ability to produce melanin. The incidence of CM has increased worldwide during the last several decades, with a higher prevalence in males and younger adults (1). It frequently arises from chronically sun-damaged skin and is characterized by a high mutational load. The genetic landscape in CM includes many different driver and passenger gene mutations implicated in tumor cell survival and proliferation (2, 3).

During melanomagenesis, tumor cells interact with components of the immune system, whose functional activity is directed at preventing melanoma progression and metastasis (4). Although lymph node metastasis and Breslow thickness are still considered negative prognostic predictors (5), the propensity of melanoma cells to invade distant tissues also depends on their interaction with cells of the tumor microenvironment (TME) and the efficiency of the immune response. The characteristics of tumor-infiltrating lymphocytes (TILs) surrounding melanoma cells influence the prognosis while their localization, composition, and density positively correlate with survival and decreased risk of metastasis (6). In this context, both CD8^+^ and CD4^+^ T-cells represent the prevalent immune infiltrating populations found nearby melanoma cells but recent studies revealed that the presence of other molecules may potentially correlate with prognosis as the loss of expression of p16, the switch of the M2/M1 polarization of macrophages and the levels of immune checkpoints including PD-1 and VISTA (V-domain Ig suppressor of T-cell activation) (7–9).

The results of immunotherapy studies in murine melanoma models have given rise to a “cancer immune surveillance hypothesis,” which postulates the continuous activity of dendritic cells (DCs) in tumor cell recognition and elimination (10). Anti-cancer immunity consists of a sequence of functional events, referred to as the immunity cycle, whose disruption allows cancer cells to overwhelm immune system control (11, 12). Among the mechanisms allowing melanoma cells to escape immune system control are the release of immune suppressive cytokines within the TME and the up-regulation of inhibitory checkpoints on T-cells (13). The defective immunity that characterizes CM depends on derangements in both the cytotoxicity of T-cells and the function of DCs. Accordingly, manipulation of the cellular components of the immune system may be a promising therapeutic strategy in CM.

The CD34^+^ progenitor cells of DCs resides in the bone marrow, where they differentiate into specialized subsets differing in their maturation, activation and co-stimulation (14). These differentiated DCs circulate in peripheral blood while migrate to lymphoid and peripheral tissues, where they regulate both innate and adaptive (15–17), but are also able to migrate toward the TME. The critical aspects of the functional activity of DCs in various cancers, including CM, are their ability to capture foreign antigens and the efficiency of cross-priming (18). Previously, DCs were considered to be either conventional or classical DCs (cDCs), providing stimulatory functions, or tolerogenic plasmacytoid DCs (pDCs) (19). However, this classification has been recently revised based on the recognition of the plasticity of these populations, whose behavior is apparently influenced by soluble factors produced by melanoma cells (20, 21). In addition to pDCs, myeloid DCs (mDCs) are now recognized to differ in their phenotype, migratory capacity and their response to chemotactic stimulation, chemokine repertoire, and morphology. The level of circulating mDCs was shown to correlate with melanoma activity and the detection of these cells in patients at high risk of recurrence may reflect the persistence of malignant cells within the pre-metastatic niche (22). However, in addition to this pathway of melanoma progression, many others have been recently explored and thus usable in immunotherapy. For example, melanoma cells may also overcome immune system control through the production of negative mediators as transforming growth factor (TGF)-β, the activation of metabolic pathways such as either indolamine 2,3-dioxygenase (IDO) or CD39/CD73 axis and, lastly, the overexpression of negative immune checkpoint receptors by T-cells and related ligands (23). This phenomenon is defined as immune exhaustion or anergy and is characterized by a progressive loss of the immune activity. To this regard, some of these negative immune checkpoints bind ligands expressed by mature DCs.

## Dendritic Cell Subsets and Their Role in Melanoma Immune Infiltrate

Dendritic cells originate from a bone marrow-derived population and circulate in blood and peripheral tissues where drive the innate immune response by processing antigens to naïve T-cells (24). Different types of specialized DCs have been described in mouse and humans, and the cellular or molecular mechanisms regulating their interaction with T-cells are at present partially known. Thus, the deep knowledge of different DC subsets requires the adoption of a dedicated nomenclature (25). In this context, a functional taxonomy distinguishes “DCs resident in lymph node” from “migratory tissue DCs” while a recent classification is codified in relation to the transcription factors' expression as interferon regulatory factor (IRF)-8, and IRF-4 (26). Therefore, pDCs and two types of cDCs have been described, the latter corresponding to the previously characterized subset of humans mDCs that also bear CD141 and CD1c antigens (27). Although cDCs and mDCs conventionally include a unique population, they substantially differ in terms of antigenic profile. Based on the most recent findings, specialized subsets of DCs may be indeed classified as pDCs, cDC1 and cDC2, Langerhans cells (LCs), monocyte-derived DCs (Mo-DCs), and small groups of cells including pre-DCs and non-classical monocytes.

Plasmacytoid DCs have been largely investigated and typically produce large amounts of interferons and cytokines, thus resulting implicated in viral infection and cancer, including CM. These cells retain the CD123, CD303, and CD304 expression while do not express myeloid antigens. They circulate in peripheral blood and migrate toward peripheral sites whose accumulation may resemble as prognostic marker in melanoma (28). Myeloid cDC1 and cDC2 show a unique antigenic repertoire but cDC1c are characterized by an intrinsic ability to present antigens through the class-I MHC for the stimulation of T-cells and both drive the Th1 polarization and promote the activation of NKs. Furthermore, human cDC1 express the chemokine CXCL9 and CXCL10 and secrete type-III interferon (IFN) and IL-12. By contrast, cDC2 are characterized by high levels of XCR1 chemokine receptor by which that permits their crosstalk in the TME with XCL-producing cells and NKs (29–31). In addition, cDC2 produce cytokines as IL-12, IL-23, IL-1, IL-8, and IL-10 while modest is the capacity to secrete type-III IFN. In mice, cDC1 have been also characterized as cells exerting tolerogenic functions but this role has not definitely proven in humans. However, cDC2 cells are the prevalent population in human blood and they express typical markers of myeloid differentiation, whereas a variable sensitivity to different transcription factors has been demonstrated in relation to their murine or human derivation. Both subsets of cDCs produce large amounts of cytokines by which they modulate the immune response. With respect to the CM microenvironment, the basal epidermis and stratified squamous epithelia are engulfed of LCs showing high levels of C-type lectin langerin as well as E-cadherin, EpCAM and claudin by which those cells closely interact with the epithelial layer (32). LCs are characterized by high migratory behavior to the skin-draining lymph nodes and are mostly accumulated nearby the T-cells enriched tumor area. Another group of DCs involved in the regulation of immune response includes those derived from monocytes (Mo-DCs) that can be identified in relation to the expression of myeloid markers and high production of IL-1, IL-12, and IL-23 (33).

In the context of melanoma, DC subsets modulate the T-cell activation and their defects are strongly correlated with clinical worsening related to the melanoma progression. Apart from the amounts of DCs that circulate in the peripheral blood, they usually surround the tumor cells in the TME forming the so-called immune infiltrate that is considered an indicator of responsiveness to immunotherapy (34). The efficacy of immunotherapy is, however, limited to a subset of patients. The anti-tumor activity of TME is exerted by T-cells but is also dependent on cDC1 and cDC2 activity, while the efficiency is correlated to the ability of these cells to present tumor-associated antigens and to produce cytokines implicated in the control of either survival or cytotoxic activity of T-cells. However, the cDC1 subset is extremely rare in humans although its levels strongly correlate with prognosis and responsiveness to immunotherapy. It has been also reported that cDC1s may expand through the transcription factor Batfl3 and express differential molecules as CD103 in murine or BDCA-3 in human melanoma (35). Further studies have demonstrated that the abundance of cDC1s surrounding tumor cells and their activity directly depend on the expression of the gene encoding Fms-related tyrosine kinase-3 ligand (FLT3LG) whose intra-tumoral production is regulated by NKs (34, 36). Moreover, the recruitment of cDC1s in TME is also driven through CCR5 signals activated by intra-tumoral CCL5 transcripts whose levels correlate with gene signature of NKs and cDC1s as well as with increased overall survival (OS). On the other hand, prostaglandin-E2 produced by melanoma cells restrains the NK-cDC1 axis by impairing their viability and the chemokine production as well as by down-regulating the expression of CCR5 on cDC1s (37–39).

In conclusion, the immune infiltrate in terms of cells and soluble factors is a relevant indicator for early identification of good responders to immunotherapy, but also suggests that other receptors expressed by immune cells could be druggable in CM.

## The Triple-E and Immune System-Melanoma Interaction: Elimination, Equilibrium and Escape

Dendritic cells are critical to the activation of the adaptive immune system, based on their ability to migrate toward peripheral sites, where they are involved in the surveillance of antigens expressed by cancer cells. Stimulated by the melanoma, DCs mature and migrate to lymphoid tissues, where they interact with effectors of the immune response, including T- and B-cells as well as natural killer cells (NKs) (17, 40). DC maturation and efficient cross-priming are regulated by the interaction of the T-cell receptor (TCR) with molecules of the major histocompatibility complex (MHC) as well as the binding of CD80/86 with the T-cell stimulator CD28 or with the negative regulator CTLA-4, mostly within the lymph nodes (41) or by cytokine-induced signals and a specific chemokine profile (42, 43).

In CM and other immunogenic cancers, the altered recognition and elimination of malignant cells by the immune system has given rise to the concept of cancer immunoediting, which groups the interactions between (44) the immune system and cancer cells into distinct elimination, equilibrium, and escape phases required for the priming of an anti-melanoma response (12).

### Elimination

During this phase, either innate or adaptive immunity attempts to eradicate the tumor cells, which are not yet clinically detectable. Effectors of the innate immune response include NKs, NK-T cells and γδ T-cells, which are responsible for TME remodeling (45) and are stimulated by interleukins (ILs) produced by melanoma cells, tumor-associated macrophages (TAMs), and stromal cells. In addition, the over-production of cytokines stimulates both the inflammatory response and the recruitment of immune cells to induce the apoptosis of melanoma cells, leading to the release of new antigens that stimulate the adaptive immune response. During this phase, both maturation and DC homing to lymph nodes occur, resulting in the enhancement of antigen presentation and the formation of tumor-specific naïve T-cells. Recruitment of these cells into the tumor bed drives the killing of melanoma cells by enhancing interferon (IFN)-γ release and the induction of apoptosis through perforin, TRAIL and Fas-L as well as the inhibition of angiogenesis. However, the inflammation that potentially takes part in the DC maturation may, conversely, facilitate the tumor progression through signals driven by IL-10 and TGF-β which are also major effectors of regulatory T-cells (Treg) development (46, 47).

### Equilibrium

Over time, cancer cells develop resistance to effector immune cells via the selection of clones with limited immunogenicity (48). This accounts for the ability of melanoma cells to survive even in an immune-competent host. Moreover, melanoma cells may harbor a number of somatic mutations that not only support their rapid proliferation, but also enhance their genetic instability, thereby favoring the development of less immunogenic clones with a strong propensity to expand within the TME (49). Thus, the equilibrium phase consists of a chronic attempt by the immune system to eliminate tumor cells, mostly by the release of high levels of IFN-γ (50).

### Escape

The majority of malignant cells are eliminated during the equilibrium phase but new variants may emerge with mutations that increase the resistance to immune system control (44) and therefore promote cancer cell proliferation. Immune system escape includes alterations in the expression of signal transduction molecules on effector cells, the production of soluble factors by melanoma cells, the induction of immune-cell tolerance, a loss of the signal transducer CD3-ζ, variability in the expression of the tumor antigen repertoire and the polarization of T helper-1 (Th1) cells (51). Among the tumor-derived soluble factors that contribute to immune cell suppression are vascular endothelial growth factor (VEGF), tumor necrosis factors (TNFs), TGF-β, IL-1, IL-6, IL-10, prostaglandin E2 (PGE2), Fas, and Fas-L. VEGF inhibits DC maturations via Stat-3 transcription and regulates both the recruitment of TAMs, T-regulatory cells (T-regs), and myeloid-derived suppressor cells (MDSCs) (52, 53). These soluble factors create a favorable environment to support tumorigenesis by increasing the cancer cell proliferation, angiogenesis, metastasis as well as exerting a direct effect on DC maturation that, at least in part, contribute to enhance the immune suppression. In this context, IL-6 is central in melanoma development and also enhances the IL-10 production via STAT-3-dependent signaling (54). Apart from interleukins, the melanoma microenvironment is also engulfed of prostanoid including PGs. Among PGs, the PGE2 plays an exclusive role in tumor development, progression and metastasis directly acting on both tumor cells and stromal compartment. However, many studies focusing the role of PGE2 in cancer are not conclusive but a clear evidence of its effect in driving tumor progression has been demonstrated in BRAFV600E mutant murine melanoma model (55, 56).

Besides soluble factors released by tumor cells, deregulated antigen processing and presentation are relevant events promoting the melanoma progression. The impaired immune response is mostly attributable to either a defective activity of DCs or the reduced cytotoxicity mediated by CD8^+^ T-cells (52, 53). In addition, the heterogeneity of the antigenic repertoire is a peculiar feature of melanoma cells and a requisite for evading the immune system control (57). Antigens are displayed to CD8^+^ cells through presentation as part of the major histocompatibility class-I (MHC-I) complex on the membranes of melanoma, immune, and accessory cells infiltrated within the microenvironment. This event is to be considered a portrait of the melanoma milieu for activated CD8^+^ T-cells. In this context, highly proliferating melanoma cells reduce the visibility of MHC-I to cytotoxic cells and many studies in cancer models, including melanoma, reported an association between loss of MHC-I activity and survival. The mechanisms favoring the MHC masking to effector T-cells include (a) genetic and epigenetic modifications; (b) altered activity of the immunoproteasome including the three subunits formed by low molecular weight polypeptides (LMPs), namely LMP-2, LMP-7, and LMP-10. The proteasome is responsible for the turnover of proteins while LMPs provide the optimal anchor residue for stable binding to MHC-I; (c) reduced activity of the transporter associated with antigen processing (TAP) whose activity consists in the efficient transport of peptides into the endoplasmic reticulum and their loading onto MHC-I complex; (d) low expression of the chaperone calnexin, calreticulin (CRT), ERp57, and the peptide editor tapasin that serves as bridge between MHC-I, TAP, and CRT. These alterations have been demonstrated in melanoma, lung, prostate, and ovarian cancer and result variably implicated in the inhibition of those processes that regulate the antigen capturing and presentation that result impaired in cancer (58, 59). In addition, tumor cells are surrounded by infiltrating lymphocytes and macrophages whereas there is compelling evidence that the amounts of immune cells infiltrating tumor cells correlate with survival in breast, colon, and pancreatic cancer as well as melanoma. Moreover, as part of the immunoediting process, the degree of intra-tumoral MHC-I down-regulation directly correlates with both type and density of infiltrating immune cells, macrophages as well as levels of IFN-γ (60–63).

## Dendritic Cells and the Melanoma Microenvironment

### Dendritic Cells and Role of TLRs

As the most potent professional antigen-presenting cells (APCs), DCs are critically involved in the capture and presentation of tumor antigen to naïve T-cells and thus in immune surveillance. The generation of an efficient immune response by DCs requires their active mobilization in response to specific gradients generated in distant tissues and their intra-nodal migration. Immune stimulation is driven by the ability of DCs to engulf cargo molecules in vesicles and exosomes, which results in the release of the molecular signals that induce immune system activity (64, 65). Among the early events required for the activation and migration of DCs are the over-expression of CC-chemokine receptor-7 (CCR7), whose binding with CCL21 guides DCs into the lymphatic vessels through haptotaxis (66, 67). CCR7 expression is under the control of the transcriptional activator nuclear factor-kB (NF-kB) (68, 69). Once inside the lymphatic vessels, DCs migrate first to the subcapsular sinus and then into the lymph node parenchyma. The lower basement membrane of the subcapsular sinus is enriched in T-cells, the recruitment of which is favored by structural modifications induced by DCs through the interplay of CCR8/CCL21 and L-selectin (70–72). The different subsets of cDCs found in the dermis include XCR1^+^, CD11b^+^ and XCR1^−^/CD11b^−^ DCs (73, 74) as well as CCR2^+^ monocyte-derived DCs.

In CM, however, impaired DC maturation, the defective expression of CD80 and CD86 co-stimulatory molecules and the up-regulation of CTLA4 or PD-1 receptors (75) result in clinical progression of the disease. Langerhans cells, a subset of APCs localized in the epidermis, are affected by melanoma progression as well, including their immune activity and number (76). While normally anchored to keratinocytes by E-cadherin, LCs acquire motility under the influence of TGF-β and thus promote melanoma progression via immune suppression, including peripheral tolerance, the expansion of Tregs and the stimulation of IL-10 production in the TME. Like cDCs, LCs respond to CCR7 by migrating to lymph nodes; however, they accumulate in the deep paracortex whereas cDCs localize to the interfollicular space.

Another critical property of DCs for the discrimination of non-self antigens is represented by pattern-recognition receptors that include the family of toll-like receptors (TLRs). Thirteen different TLRs have been described in mammals although only 10 out of them are encoded in the human genome (TLR1-10). An implication of TLRs in DC biology includes their activity as defensive barrier by the recognition of microbial components as microbe, pathogen-associated molecular patterns (MAMPs, PAMPs), and damage-associated molecule patterns (DAMPs) (77). In addition, the DC interaction with TLR ligands induces the production of type-I IFNs and other pro-inflammatory cytokines within the TME. This interplay also promotes the DC maturation and enhances their antigen presentation ability to naïve T-cells, thus resulting in effective activation of the adaptive immunity.

However, the pattern of TLR expression differs among the DC subsets. In fact, pDCs mostly express both TLR7 and TLR9, whereas cDCs are TLR1-7^+^ but negative for TLR9. Moreover, these cells produce several cytokines in response to TLR ligands and, as previously demonstrated, high levels of type-I IFNs are produced by pDCs stimulated with TLR7 or TLR9 ligands, while cDCs release IL-12 following the interaction with TLR7, TLR8, and TLR4 ligands. Indeed, further evidence suggest that the stimulation of TLR2^+^ DCs by tumor-derived TLR2 ligands drives inhibitory signals leading to dysfunctional activity of DCs in murine melanoma (78). On the other hand, activation of TLR2^+^ DCs inhibits the immune responsiveness of TME through the release of IL-10 and IL-6, while DC themselves may suffer of an autocrine negative effect exerted by the upregulation of both IL-6 and IL-10 receptors. Further studies have shown that TLR2 enhances the immune suppression by stimulating the MDSCs survival, thereby restraining the anti-tumor T-cell activity (79).

The increasing knowledge regarding the effects mediated by the activation of TLRs on DCs is a requisite for the development of TLR agonists. In this context, they have been investigated in pre-clinical models and clinical trials, thus revealing a potential role as adjuvants for the anti-cancer immunotherapy (80). In this context, Imiquimod is a TLR-7 agonist used for the treatment of skin disorders including basal cell carcinoma. It shows structural similarity with the adenosine nucleotide analog and thus may inhibit the Hedgehog signaling by stimulating the adenosine receptor. In addition, the Bacillus Calmette-Guerin (BCG) is a well-known TLR agonist exerting activity on TLR2, TLR4, and TLR9 in bladder cancer. These findings have leaded the basis for the development of many translational and pre-clinical studies in different cancer models including metastatic melanoma. In particular, a TLR3 agonist has been used in patients suffering of myeloid acute leukemia leading to a functional restoration of the immune system activity. Moreover, a TLR4 agonist (GSK1795091) has been tested in phase-I trial, whereas Imiquimod followed by bevacizumab received the FDA (Food and Drug Administration) approval in refractory glioblastoma. Other studies demonstrated that the combination of a TLR3 agonist (ARNAX) with a PD-L1 blocker might overcome the resistance to anti-PD-1 treatment (75). To this regard, a promising role is played by TLR9 agonists (CMP-001, SD-101) that have been recently investigated in refractory metastatic melanoma patients for overcoming the acquired resistance developed to anti-PD1 blockers, thus leading to local and systemic effects or turning the immunologically “cold” to “hot” tumor (80). Furthermore, basic researches suggested the interplay of TLR-mediated signaling with the local metabolism activated in the TME and that the block of TLR7 and TLR8 signals drives a NK cell-dependent immune response against tumor cells (75). Based on the preliminary results achieved by agonist of TLR9 in metastatic melanoma, a phase-3 study (IMO-2125) is ongoing while the experimental arm is exploring the effect of Tilsotolimod in combination with ipilimumab in restoring the immunostimulatory activity, the DC activation, the CD8^+^ cell proliferation as well as a potential abscopal effect (81).

### Tumor Microenvironment and Immune Metabolism

During the metastatic phase, melanoma cells orchestrate the establishment of an immune suppressive TME, by stimulating neo-angiogenesis, escaping from T-cell recognition and developing resistance to radio/chemotherapy (82). The acquisition of these properties involves extracellular matrix remodeling, cytokine over-production, the recruitment of inhibitory cells and the intrinsic genetic mutations harbored by melanoma cells. Because an efficient immune response depends on the availability of nutrients and bioenergetics (83, 84), the high metabolic demand of the proliferating tumor cells and the formation of a stromal network overexposes T-cells to suppressive metabolites, which results in T-cell exhaustion and a reduction in the efficiency of their effector function (85). This metabolic reprogramming that involves immune cells during their activation has been recently defined as immunometabolism (85). In this context, the adenosine signaling that involves the CD39/C73 ectonucleotidases is a critical pathway used by melanoma cells to generate an immunosuppressive milieu (86). Indeed, the extracellular adenosine is the result of CD39 enzyme activation that induces the dephosphorylation of the adenosine triphosphate (ATP) to adenosine disphosphate (ADP) and then to adenosine monophosphate (AMP). Lastly, CD73 catalyzes the hydrolysis of AMP into phosphate and adenosine that suppresses the TME, thus establishing a “purinergic milieu.” On the other hand, the CD39/CD73/adenosine signaling establishes an immunosuppressive melanoma microenvironment restraining both innate and adaptive anti-tumor immunity through dedicated adenosine receptors, namely A1, A2A, A2B, and A3 (87). These effects are variably modulated by the adenosine receptors while the A2A is the most common that outlines inhibitory signals by decreasing the activity of T-cells, NKs, NK-T cells, macrophages, and DCs as well as enhancing the recruitment of Treg and MDSCs in the TME (88). Moreover, studies completed in murine melanoma suggest that the A2B receptor promotes the CD11b/Gr1^+^ MDSCs accumulation in tissues and lymph nodes nearby melanoma cells (89). With regard of DC function, adenosine has been described as a critical modulator of their activity during the early cross-priming presentation process as well as IL-10 production and inhibition of CXCL10 release.

The concomitant IL-10 production from DCs associated with low levels of IL-12 within the TME provokes the recruitment of Treg. Similarly, adenosine also inhibits the IL-12 and IL-6 cytokine production by pDCs, thus interfering with immune response in viral infections, autoimmunity and cancer (90). Moreover, adenosine induces a defective maturation and differentiation of DCs toward a pro-angiogenic and tolerogenic phenotype, thus allowing tumor cells to escape the immune surveillance. In conclusion, adenosine produced by DCs suppresses the cross-priming presentation thus prompting the T-cell anergy while the adenosine released in TME inhibits the maturation and differentiation of DCs.

In addition, nicotinamide adenine dinucleotide (NAD) pathway also concurs to the immunometabolism dysfunction. NAD is a co-enzyme that controls the redox reactions in several metabolic pathways, including glycolysis through the nicotinamide phosphor-ribosyltransferase (NAMPT), a rate-limiting enzyme for the NAD synthesis frequently overexpressed in cancer cells (91). Moreover, NAMPT-specific inhibitors may deplete the NAD levels and restrain the cancer cell proliferation by inhibition of the energy production (92).

Another emerging inhibitory enzyme involved in the immunometabolism pathway is IDO, which is expressed by both melanoma cells and DCs, and mediates the conversion of tryptophan into kynurenines, thus inducing the local depletion of essential amino acids and restraining the recruitment of CD8^+^ T-cells in favor of both Treg and MDSCs. Both cell types produce inhibitory cytokines as well as arginase-1, nitric oxide synthase, and reactive oxygen species (93). In CM patients, Treg levels were shown to correlate with clinical activity, thus demonstrating the utility of immune cell detection in the monitoring of melanoma progression, while MDSCs promote the differentiation of CD4^+^ T-cells into CD4^+^/CD25^+^/Foxp3^+^ cells, which express TGF-β and IL-10 as well as CTLA-4 and PD-1. The binding of these latter receptors to ligands expressed on DCs limits the crosstalk with CD8^+^ T-cells. Consequently, the peripheral and intra-tumoral expansion of CD4^+^/CD25^+^/Foxp3^+^ cells correlates directly with melanoma progression.

### Characteristics of the Immune Infiltrate

**Figure 1:**
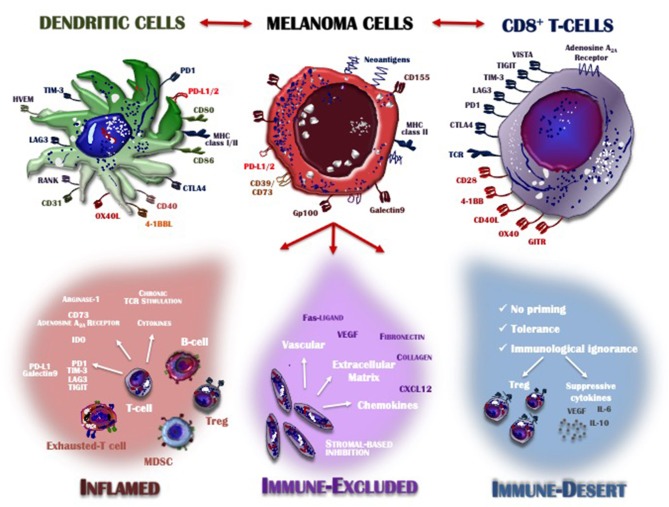
Inflamed vs. non-inflamed tumor microenvironment. The melanoma microenvironment consists of a complex immune infiltrate consisting of dendritic cells (DCs), CD8^+^ T-cells, natural killer cells (NKs), regulatory T-cells (Treg), myeloid-derived suppressor cells (MDSCs), cytokines, enzymes, and negative modulators. Apart from melanoma cells characterized by the loss of HLA-I expression and thus already capable of evading the immune system control, the infiltrate assumes three different phenotypes: inflamed (red), immune-excluded (violet), and the immune desert (blue), depending on whether its features contribute to overcoming or supporting anti-tumor immunity control. Tumors with an immune-inflamed profile are highly responsive to immunotherapy but their rich immune infiltrate includes dysfunctional T-cells exhausted by chronic antigen exposure. Immune-excluded tumors are characterized by a pre-existing anti-melanoma response and specific stromal-based inhibition whereas immune-desert tumors are immunologically “ignorant,” as T-cells rarely reach the tumor parenchyma or stroma. Melanoma cells, DCs and T-cells cross-talk through inhibitory or activating receptors, by which the immune response is induced or restrained, thus creating an equilibrium aimed at limiting melanoma cell proliferation and metastasis. MHC, major histocompatibility complex; CTLA-4, cytotoxic T-Lymphocyte antigen 4; PD-1, programmed death-1; PD-L1/2, programmed death-ligand 1/2; IDO, indoleamine 2,3-dioxygenase; TIM3, T-cell immunoglobulin mucin 3; LAG3, lymphocyte-activation gene 3; TIGIT, T cell Ig and ITIM domain; VISTA, V-domain Ig suppressor of T cell activation; TCR, T-cell receptor; MDSC, myeloid-derived suppressor cell; Treg, regulatory T cell; VEGF, vascular endothelial growth factor; IL, interleukin; CXCL, C-X-C motif ligand; GITR, glucocorticoid-induced TNFR-related protein; HVEM, herpes virus entry mediator; RANK, receptor activator of nuclear factor kappa-B.

Detailed knowledge of many aspects of the immune system together with the results achieved by immunotherapy has provided insights into the features of the T-cells that infiltrate the TME. The recruitment of immune cells is mostly regulated by the selectins, adhesion molecules and integrins expressed on melanoma cells and by the expression of CXCR3 chemokine receptor on T-cells and its ligands CXCL9, CXCL10, and CXCL11, which are key immune chemoattractants during interferon-induced inflammatory response (94). Based on the characteristics of the immune infiltrate, the cancer microenvironment may assume one of three phenotypes (Figure 1): the immune desert, the immune-excluded, and the inflamed phenotype. Their cellular and molecular properties are pivotal determinants of both melanoma progression and also therapeutic response (57).

The immune desert phenotype is the consequence of immunological ignorance, based on both the activation of tolerance and inappropriate priming. The anti-melanoma response to tumor-associated antigens and neo-antigen formation is therefore limited and antigen presentation inadequate. Other features of the immune desert milieu reflect the absence of pre-existing anti-tumor immunity, evidenced by modest T-cell helper activity, an insufficient number of T-cells and the recruitment to lymph nodes of immature and poorly co-stimulated DCs (95). In the immune-excluded phenotype, immune cells infiltrate the stroma surrounding the tumor cells but interact only minimally or not at all with the tumor itself. Thus, although the immune-excluded TME suggests a pre-existing anti-melanoma response, it seems to have been rendered ineffective by the inability of the immune cells to access the tumor (96). Based on similar observations in murine melanoma models, the primary role of CD103^+^ DCs seems to be the regulation of T-cell entry into the tumor mass (97). Both the immune desert and the immune-excluded phenotypes represent a non-inflamed TME. In the inflamed TME, the stromal tissue surrounding the melanoma cells is enriched in CD4^+^ and CD8^+^ cells but they are hyper-exhausted. In addition, accessory cells such as monocytes, myeloid cells and Tregs are frequently detected. The tumor cells produce large amounts of IDO and activating cytokines, such as IFNs, IL-12, and IL-23, while over-expressing PD-L1-driven inhibitory signals, down-regulating MHC class I molecules and activating alternative pathways that weaken anti-cancer immunity (97). Thus, the inflamed profile suggests a pre-existing anti-tumor response that is arrested by immune suppression in the tumor bed. However, these cancers are also highly responsive to anti-PD-1 immunotherapy.

## The Role of Inhibitory Immune Checkpoints in DC-Mediated Anti-Cancer Immunity

In melanoma, DCs provide the immune bridge linking innate and adaptive anti-tumor immunity (12). The DC maturation involves several well-studied co-stimulatory molecules. Besides, co-inhibitory receptors, most of which remain undefined, also mediate the DC maturation and function. Interestingly, the interplay of inhibitory signals in DC crosstalk with both tumor cells and activating or regulatory immune cells, modulates the effectiveness of DC-mediated anti-cancer immunity, thus shaping the TME (Table 1).

**Table 1:** Key molecules and receptors implicated in the DC/melanoma interplay.

**Receptor(s)/ligand(s)**	**Effect(s) on lymph nodes/TME**	**References**
MHC-class I/II	Initiation of the antigen-specific immune response, antigen processing and presentation, and the cross-priming processes	(17)
B7-1 (CD80)/B7-2 (CD86)	Modulation of DCs co-stimulation and cross-priming interference based on activating/inhibitory receptor expression	(98)
OX40 (CD134) OX40L (CD252)	Induction of maturation, activation, and survival of DCs	(99)
CD40 (CD154) CD40L	Co-stimulatory molecule that play a central role in B and T-cell activation	(75)
CD28	“Secondary signal:” co-stimulation of DCs inducing the complete activation and effector functions in T-cells	(100)
4-1BB (CD137) 4-1BBL	CD137 ligand signaling induces human monocyte to dendritic cell differentiation	(101)
CTLA4 (CD152)	CTLA4 exerts an inhibitory role in mature DCs through the autocrine uptake of vesicles enriched of CTLA4 molecules that restrain the co-stimulation	(102)
PD1	Suppression of CD8^+^ T-cell activity and decrease of T-cell infiltration	(103)
PD-L1 (CD274)/PD-L2	Inhibition of CD4^+^ and CD8^+^ T-cell activity; promotion of Tregs expansion; abrogation of NKs function.	(104)
TIM-3	Induction of apoptosis of Th1 polarized T-cells	(105)
LAG3	LAG-3 is constitutively expressed on pDCs, playing an important role in both the homeostatic maintenance and activation-induced expansion of pDCs	(106)
BTLA/HVEM	Induction of Treg differentiation, up-regulating the expression of CD5 by T-cells	(107)
CD31 (PECAM-1)	Upholding CD31 signaling during maturation converts stimulated DCs in TME into tolerogenic cells	(108)
RANK	Induction of maturation, activation and survival of DCs	(109)

*TME, Tumor microenvironment; MHC, Major histocompatibility complex; CTLA4, Cytotoxic T-Lymphocyte Antigen 4; DCs, Dendritic cells; NKs, Natural killer cells; HVEM, Herpes virus entry mediator; pDCs, Plasmacytoid dendritic cells; RANK, Receptor activator of nuclear factor kappa-B*.

T-cell activation is a key event in the adaptive immune response and thus in the generation of a cell-mediated anti-tumor effect. Early T-cell activation is regulated by activating and inhibitory receptors, including CD28 and CTLA4, whose interplay results in integrated intracellular transcriptional signals that balance T-cell activation and self-tolerance. Another important step is the appropriate activation of effector CD4^+^ and CD8^+^ T-cells, which requires the engagement of MHC molecules expressed by DCs with the TCR, followed by the generation of a first signal consisting of the TCR/CD3 complex (110). TCR binding to antigen-loaded MHC is followed by a secondary signal that is driven by the ligands CD80 (B7-1) and CD86 (B7-2). These molecules act as co-stimulators via CD28 (98), which is highly expressed upon activation with CD80, and binds the inhibitory protein CTLA4 (102) largely exposed by T-cells and especially Tregs. CTLA4 shows homology with CD28 (42, 100) and its expression by mature DCs (104, 111) inhibits their own maturation through the autocrine uptake of CTLA4-enriched vesicles (112).

A relevant breakthrough in melanoma has been obtained by immunotherapy combinations, revealing that the CTLA4 activation mostly occurs in lymph nodes while the modulation of T-cell functions by PD-1 stimulation has been mostly described in the microenvironment of peripheral tissues (113). The PD-1 engagement by related ligands, PD-L1 (B7-H1) and PD-L2 (B7-DC) that are differently expressed by tumor and immune cells, is known to recruit phosphatase SHP-1 and SHP-s to specific ITIM (immune-receptor tyrosine-based inhibitory motif) and ITSM (immune receptor tyrosine-based switch motif) cytosolic loci, thus inducing the de-phosphorylation of proximal TCR signaling that concurs to suppress both mTOR and PI3K/AKT intracellular pathways. In contrast to the restricted levels of PD-L2 on activated macrophages, PD-L1 is predominantly expressed on non-hematopoietic and immune cells. PD-L1 is constitutively present on B cells, macrophages, T-cells and DCs and is upregulated after the stimulation of pro-inflammatory cytokines such as IFN. Moreover, it has been also described a “constitutive” oncogenic signaling able to over-expresses PD-L1 ligand on tumor cells, regardless of inflammatory signals in the TME (101). This supports the notion that PD-L1 acts as molecular cloak on both host immune and tumor cells to protect cancer from cytolysis by T-cells.

Therefore, the PD-1 interaction with both PD-L1 and PD-L2 promotes the T-cell dysfunction, exhaustion, and anergy (101). Apart from the interaction between T lymphocytes and tumor cells, the engagement of the PD-1/PD-L1 axis also occurs among immune cell types infiltrating the TME. To this regard, DC-mediated anti-tumor immunity driven by the PD-1/PD-L1 axis involves different mechanisms, as the cells express either the receptor or the ligands that interact with PD-1^+^/PD-L1^+^/2^+^ cells. PD-1 expression by mDCs and pDCs is up-regulated by pro-inflammatory cytokines that have accumulated in the TME (114, 115) while PD-L1^+^ DCs expand the CD4^+^/CD25^+^/FoxP3^+^ Treg population, leading to immune suppression (116). The role of the PD1/PD-L1 axis in cross-talk between DCs and other innate immune cells, including NKs, NK-T cells, γδT-cells, regulatory MDSCs and TAMs, is still unknown. However, inhibition of the PD-1/PD-L1 axis was shown to reinforce the NK activity in multiple myeloma (105, 107, 117), and in a pre-clinical melanoma model, the up-regulation of PD-L1 by tumor-infiltrating and tumor-draining lymph nodes resulted in decreased PD-1^+^ DC cross-priming activity (118). In addition to CTLA4 and PD-1 inhibitory receptors, other emerging immune checkpoint molecules (Figure 1) involved in DC-mediated immunity include TIM-3 (T cell immunoglobulin and mucin domain-3), BTLA, LAG3, and CD31 (119, 120). The role of TIM-3^+^ T-cells in the modulation of the TME has been largely investigated in parallel with the membrane expression of this receptor by tumor-associated DCs (108). TIM3^+^ DCs promote the apoptosis of Th1 T-cells subsequent to receptor interaction with the galectin-9 ligand (121). In a mouse model of fibrosarcoma, the dual inhibition of TIM-3 and PD-1 or CTLA4 reduced tumor growth (122). BTLA is an immunoglobulin domain superfamily protein that controls the expression of CD5 by T-cells via engagement of the ligand HVEM (herpes virus entry mediator), which results in MEK phosphorylation and, in turn, Treg differentiation and peripheral tolerance (109). CD31 forms a co-receptor with PECAM-1 that is constitutively expressed by resting DCs (123, 124). Recent studies have demonstrated that CD31/PECAM-1 engagement by T-cells balances their activation and tolerance whereas co-receptor inhibition favors the maturation and migration of resident DCs to draining lymph nodes (125).

Although the RANK-L/RANK axis represents a key regulator of the bone remodeling and critical for the osteoclastogenesis machinery, RANK-L also drives the DC differentiation and survival (126, 127). Indeed, the reciprocal interaction between T-cells and DCs through RANKL/RANK system induces both NF-kB activation and pro-inflammatory cytokines release, thus providing the DC survival and viability (128). By contrast, an immune suppressive alternative activity of the RANKL/RANK axis in TME has been described because RANKL induces activation of RANK on melanoma-associated DCs thus exerting a tolerogenic effect (129).

## Dendritic Cells and Personalized Therapy in Melanoma

Besides immune checkpoint receptor expression by DCs, the mechanisms that contribute to inhibiting the tumor milieu include metabolic reprogramming, such as induced by the extracellular release of adenosine. In fact, recent data have demonstrated that A2A adenosine receptor blockade by a selective antagonist or CD73 inhibitor reinforces the efficacy of DC-based vaccination (130). The therapeutic potential of DC vaccination may likewise be strengthened by a blockade of the pioneering CD28/CTLA4 and PD1/PD-L1 pathways. Other studies have shown that the efficacy of vaccination is highly likely in tumors bearing a low tumor mutational burden, whereas a therapy based on the blockade of immune checkpoints may have greater success in patients with immunogenic melanoma characterized by a high TMB (47, 131).

However, checkpoint inhibitors poorly stimulate the immune response in patients with tumors surrounded by a limited T-cell infiltrate (132). The DCs that co-localize in the peri-tumoral infiltrate consist of two specialized subsets: CD103^+^/CD8^+^ DCs, characterized by excellent priming and cross-presentation activity, and CD11b^+^ DCs, which promote the activity of CD4^+^ T-helper cells (96). Nonetheless, molecular investigations in human metastatic melanoma specimens revealed a correlation between the activation of the Wnt/β-catenin signaling and resistance to anti-PD-1 and anti-CTLA-4 monoclonal antibodies (133). Moreover, defective activation of CCL4 chemokine was shown to result in the reduced recruitment of CD103^+^ DCs. The populations of immune cells infiltrating the tumor bed in melanoma and delivering intact antigens to tumor-draining lymph nodes have been extensively studied in murine models of melanoma and in humans (134).

**Figure 2:**
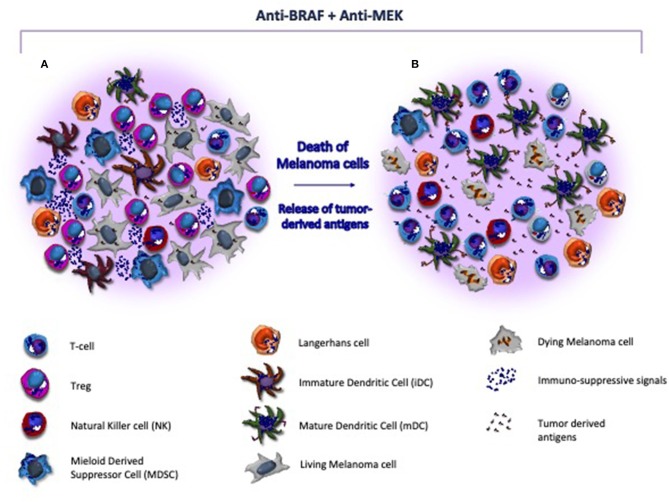
Target therapy restores the immune system activity in melanoma microenvironment. **(A)** Melanoma microenvironment is characterized by the paucity of cytotoxic T-cells and the prevalence of immune suppressive cells (e.g., Treg and MDSC) and soluble factors (e.g., interleukins) that favor the tumor progression. Also, DCs are blocked at an immature stage, thus resulting unable to properly present and process tumor-derived antigens to immune competent populations. **(B)** BRAF/MEK inhibitors exert direct anti-melanoma activity and restore the tumor immunogenicity within the microenvironment. As effect of targeted therapy, melanoma cells undergo to apoptosis, release neo-antigens, and hamper the immunosuppressive signals, thus restoring antigen presentation by DCs and T-cell mediated cytotoxicity. In addition, MHC-I is re-activated and both T-cells and NK cells are recruited nearby tumor, while Tregs and MDSC become impaired.

Other promising therapeutic strategies in metastatic CM include BRAF/MEK inhibitors with the potential to restore immune system properties by increasing the recruitment of CD8^+^ T-cells (Figure 2), their infiltration of the tumor and the release of tumor antigens avidly recognized by cytotoxic cells (135). Also, the innate immunity system may be, however, affected by BRAF/MEK inhibitors. In this context, the blockade of MAPK signaling was shown to inhibit the negative effect exerted by melanoma cells in terms of DC differentiation, cytokine production, antigen cross-priming and capture, thus renewing the functions of these cells (136, 137). A number of reports demonstrated that the blockade of MAPK pathway restores the anti-melanoma immunity by up-regulating the co-stimulation in terms of CD83, CD80, and CD86 expression by DCs (136). Also, NKs are influenced by BRAF/MEK inhibitors that induce the ERK1/2 phosphorylation, the upregulation of CD69, the IFN-γ secretion thus restoring their cytotoxic activity (138). Furthermore, other immune modulatory effects induced by these therapies include the impairment of MDSCs and Tregs activity, the reduced production of IL-10, IL-6, and VEGF by melanoma cells in TME as well as the down-modulation of the C-C chemokine ligand (CCL)-2 that is mostly involved in macrophage recruitment and survival (106, 139, 140).

## Conclusions and Future Perspectives

The biology of melanoma cells is closely associated with immune system regulation. As matter of fact, the relevance of DCs has been largely accepted as a needful immune population that connects innate and adaptive immune system, although their definite role requires further knowledge. Interestingly, the DC modulation in the melanoma microenvironment shapes both tumor development and anti-melanoma immunity. Therefore, DCs result as an attractive target for the manipulation of the immune system for therapeutic purposes aimed to enhance the immune response or alternatively to overcome the onset of acquired resistance to cancer immunotherapy. However, the complexity of the DCs system requires a thoughtful and rational manipulation of DCs to obtain protective or therapeutic immunity.

Moreover, why other immune cells infiltrating the tumor bed may either promote or block melanoma progression remains unclear, as does the discrepancy between the quality of the immune infiltrate and the clinical outcome in the majority of CM patients (141). Consequently, to achieve the better management of patients, the focus has shifted to the immune component of the TME. A recently developed immunoscore used in melanoma (142) and in colon cancer (143) has demonstrated that, in the TME, not only the functional features of T-cells but also their spatial distribution, density and expression of PD-L1 are crucial determinants of the response to immunotherapy. In addition, an index based on multiparametric analyses has been created to better define the spatial relationship at the invasive margin of the tumor and its stromal components. Further efforts at understanding the complex machinery that underlies the interplay between immune cells and melanoma are needed to design effective immunotherapies and to identify biomarkers that predict tumor responsiveness.

## Author Contributions

All authors listed have made a substantial, direct and intellectual contribution to the work, and approved it for publication.

### Conflict of Interest

The authors declare that the research was conducted in the absence of any commercial or financial relationships that could be construed as a potential conflict of interest.
